# Scaling the leaf length-times-width equation to predict total leaf area of shoots

**DOI:** 10.1093/aob/mcac043

**Published:** 2022-03-30

**Authors:** Kohei Koyama, Duncan D Smith

**Affiliations:** Department of Agro-environmental Science, Obihiro University of Agriculture and Veterinary Medicine, Inadacho, Obihiro, Hokkaido, Japan; Department of Botany, University of Wisconsin—Madison, 430 Lincoln Dr., Madison, WI, USA

**Keywords:** Allometry, scaling, leaf size, shoot size, intraspecific, Corner’s rule, self-affine, *Cardiocrinum cordatum*, *Fallopia sachalinensis*, *Magnolia kobus*, *Prunus sargentii*, *Ulmus davidiana* var. *japonica*

## Abstract

**Background and Aims:**

An individual plant consists of different-sized shoots, each of which consists of different-sized leaves. To predict plant-level physiological responses from the responses of individual leaves, modelling this within-shoot leaf size variation is necessary. Within-plant leaf trait variation has been well investigated in canopy photosynthesis models but less so in plant allometry. Therefore, integration of these two different approaches is needed.

**Methods:**

We focused on an established leaf-level relationship that the area of an individual leaf lamina is proportional to the product of its length and width. The geometric interpretation of this equation is that different-sized leaf laminas from a single species share the same basic form. Based on this shared basic form, we synthesized a new length-times-width equation predicting total shoot leaf area from the collective dimensions of leaves that comprise a shoot. Furthermore, we showed that several previously established empirical relationships, including the allometric relationships between total shoot leaf area, maximum individual leaf length within the shoot and total leaf number of the shoot, can be unified under the same geometric argument. We tested the model predictions using five species, all of which have simple leaves, selected from diverse taxa (Magnoliids, monocots and eudicots) and from different growth forms (trees, erect herbs and rosette herbs).

**Key Results:**

For all five species, the length-times-width equation explained within-species variation of total leaf area of a shoot with high accuracy (*R*^2^ > 0.994). These strong relationships existed despite leaf dimensions scaling very differently between species. We also found good support for all derived predictions from the model (*R*^2^ > 0.85).

**Conclusions:**

Our model can be incorporated to improve previous models of allometry that do not consider within-shoot size variation of individual leaves, providing a cross-scale linkage between individual leaf-size variation and shoot-size variation.

## Introduction

Plants are modular organisms, and they can be considered as a population of leaves and stems (Harper and Bell, 1979). Within each plant, organs (e.g. leaf or stem) usually differ in size, physiology and microenvironments (Field, 1983; DeJong *et al.*, 1989; Koyama and Kikuzawa, 2010; Niinemets, 2016; Kusi and Karsai, 2020; Maslova *et al.*, 2021). Therefore, photosynthesis of individual plants or ecosystems has been modelled as the sum of those of individual leaves (Bazzaz and Harper, 1977; Field, 1983; Ackerly and Bazzaz, 1995; Koyama and Kikuzawa, 2009). This cross-scale relationship between organ-level and plant- or ecosystem-level physiology has long been recognized as one of the central issues in canopy photosynthesis models (Field, 1991; Hikosaka *et al.*, 2016; Niinemets, 2016).

However, despite its importance, within-canopy or within-plant variation of organs has rarely been incorporated in the field of plant allometry. Allometry (i.e. power functions) has been a successful tool for analysing relationships between the properties of different-sized individual plants or organs (Niklas, 1994; Enquist *et al.*, 2009; Mori *et al.*, 2010; Savage *et al.*, 2010; Bentley *et al.*, 2013; Okie, 2013; Banavar *et al.*, 2014; Huang *et al.*, 2019; Lin *et al.*, 2020; Olson *et al.*, 2020; Kurosawa *et al.*, 2021; Wang *et al.*, 2021). However, most plant-level allometric models are based on the simplifying assumption that each individual plant has terminal organs (twigs or leaves) of the same size (Enquist *et al.*, 2009; West *et al.*, 2009; Savage *et al.*, 2010; Banavar *et al.*, 2014). These approaches contrast with organ-level studies on the within-plant size variation of twigs and leaves (Dombroskie and Aarssen, 2012; Koyama *et al*., 2012, 2017; Kusi and Karsai, 2020; Maslova *et al.*, 2021). The integration of these two approaches, plant allometry and canopy photosynthesis models, has not been achieved yet, although both approaches independently predict plant- or ecosystem-level metabolism (Koyama *et al.*, 2017).

Here, a shoot is defined as a terminal single current-year stem with all its appendages (leaves, buds, flowers, fruits, etc.). A shoot is equivalent to an individual ramet (i.e. whole above-ground part of a plant) in single-stem herbaceous species. For trees, a shoot is a fundamental unit of growth (Sterck *et al.*, 2005; Sterck and Schieving, 2007; Lecigne *et al.*, 2021) and reproduction (Chen *et al.*, 2009; Scott and Aarssen, 2013; Miranda *et al.*, 2019; Fajardo *et al.*, 2020). Given its importance, allometric relationships of shoot size and total shoot leaf area have been important topics in plant ecophysiology (Corner, 1949; White, 1983; Ackerly and Donoghue, 1998; Brouat *et al.*, 1998; Westoby and Wright, 2003; Kleiman and Aarssen, 2007; Olson *et al*., 2009, 2018; Sun *et al*., 2010, 2020; Yan *et al.*, 2013; Trueba *et al.*, 2016; Fan *et al.*, 2017; Smith *et al.*, 2017; Zhu *et al.*, 2019; Fajardo *et al.*, 2020 ). However, most previous studies on leaf vs. shoot size allometry have focused on the relationship among shoot size, total shoot leaf area, total leaf number and/or mean individual leaf size on the shoot. These studies are not mutually exclusive of, but do not yet have a theoretical connection with, the fact mentioned above that a shoot has a population of leaves with a size distribution (see Bazzaz and Harper, 1977). Because the total leaf area of a shoot (or a plant) is the sum of the areas of individual leaves, the leaf size distribution within a shoot is one of the main determinants of whole-plant or total shoot leaf area (Seleznyova and Greer, 2001; Bultynck *et al.*, 2004). Yet, with only a few exceptions (e.g. Koyama *et al.*, 2012; Smith *et al.*, 2017), this fact was not considered in most previous studies on leaf size – shoot size allometry (e.g. Sun *et al*., 2006, 2010, 2017, 2019*a*, *b*, 2020; Kleiman and Aarssen, 2007; Ogawa, 2008; Yang *et al*., 2008, 2009, 2010; Milla, 2009; Xiang *et al.*, 2009*a*, 2010; Whitman and Aarssen, 2010; Dombroskie and Aarssen, 2012; Scott and Aarssen, 2012, 2013; Yan *et al.*, 2013; Dombroskie *et al.*, 2016; Trueba *et al.*, 2016; Olson *et al.*, 2018; Miranda *et al.*, 2019; Zhu *et al.*, 2019; Fajardo *et al.*, 2020).

Therefore, the objective of this study was to clarify the relationship between size variations at two different levels: the within-species size variation of shoots and the within-shoot size variation of leaves. We propose a simple geometric model that incorporates these two size variations. The model is a mathematical quantification and generalization of the results of Koyama *et al.* (2012), which showed that differently sized plants of the herbaceous species *Cardiocrinum cordatum* share the same basic structure. However, their study did not provide a mathematical model that could derive these relationships. Furthermore, the present model is more general than the findings of Koyama *et al.* (2012), in that it can be applied to various plant forms (trees, rosettes and erect herbs). In the present model, maximum leaf size within a shoot plays a pivotal role. In relation to this, Sun *et al*. (2019*a*, 2020) recently proposed a model that unified previous studies on the leaf size–number trade-off (Kleiman and Aarssen, 2007), shoot photosynthesis and growth (Niklas and Enquist, 2001, 2002), and stem cross-sectional area [i.e. pipe model (Shinozaki *et al.*, 1964; Brouat *et al.*, 1998)]. Sun *et al*. (2019*a*, 2020) also found that maximum leaf size within a shoot is a major determinant of the leaf number per stem mass across different species. Moreover, Lopes and Pinto (2005), and Heerema, Spann, and their colleagues (Heerema *et al.*, 2008; Spann and Heerema, 2010) proposed empirical relationships that use maximum leaf size to predict total shoot leaf area. Nonetheless, all of these previous findings, specifically on the usefulness of maximum leaf size, are phenomenological because they do not provide any quantitative model to explain why maximum leaf size is a predictor of the total leaf area of a shoot. Here, we used an entirely novel approach, which uses maximum leaf size to model within-shoot and between-shoot leaf size variations.

## MODEL

Individual leaf area (*A*_leaf_) is defined as the area of one side of each lamina (i.e. leaf blade) (John *et al.*, 2017). A shoot may have one or multiple leaves, each of which may differ in size. Therefore, the total leaf area of a shoot (*A*_shoot_) is defined as the sum of *A*_leaf_ of all the leaves on that shoot:


$$\begin{array}{l} A_{shoot}\equiv \sum_{shoot}A_{leaf} \end{array}$$ (1)

The symbol ‘≡’ indicates ‘defined as’. As our aim was to find simple formulas that predict *A*_shoot_, taking into consideration the within-shoot size variation of *A*_leaf_, the present model is based on several simplifications. (1) We focused only on the leaf laminas that determine *A*_shoot_. We thus ignored any other organs (e.g. stem, petioles, buds and reproductive organs). (2) Our model only deals with simple leaves with flat-shaped laminas: the current model cannot be applied to leaves of different forms (e.g. compound leaves that consist of multiple leaflets, succulent leaves or conifer needles). The limitations associated with these simplifications will be addressed in the Discussion.

**Fig. 1.**
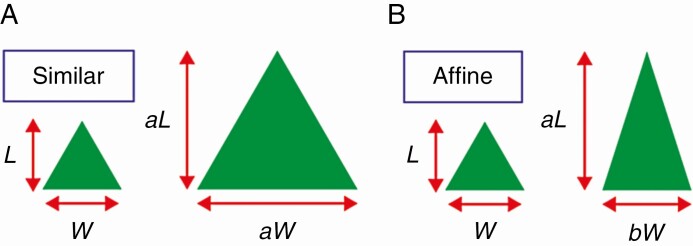
Definition of the words ‘similar’ and ‘affine’ used in this article. (A) Two similar triangles share the same length-to-width ratio. (B) Two affine triangles may have different length-to-width ratios. For an affine transformation to change the small triangle into the large triangle, the scaling factor in one direction (*a*) is not necessarily equal to that in the other direction (*b*), and similar transformation is a special case of affine transformation when *a* = *b*. In both cases, the area is proportional to the product of the length (*L*) and width (*W*).

We use the two words ‘similar’ and ‘affine’ (Fig. 1), which have been used as compound words ‘self-similar’ and ‘self-affine’ in fractal geometry (Falconer, 2003; Okie, 2013; Shi *et al.*, 2021*b*). In Fig. 1, in each panel (A and B), the two green triangles represent two different-sized individual leaf laminas. Two shapes are similar if they can be made identical by multiplying each dimension by a single constant (i.e. similar transformation). Two shapes are affine if they can be made identical by multiplying each dimension by a different constant (i.e. affine transformation).

**Fig. 2.**
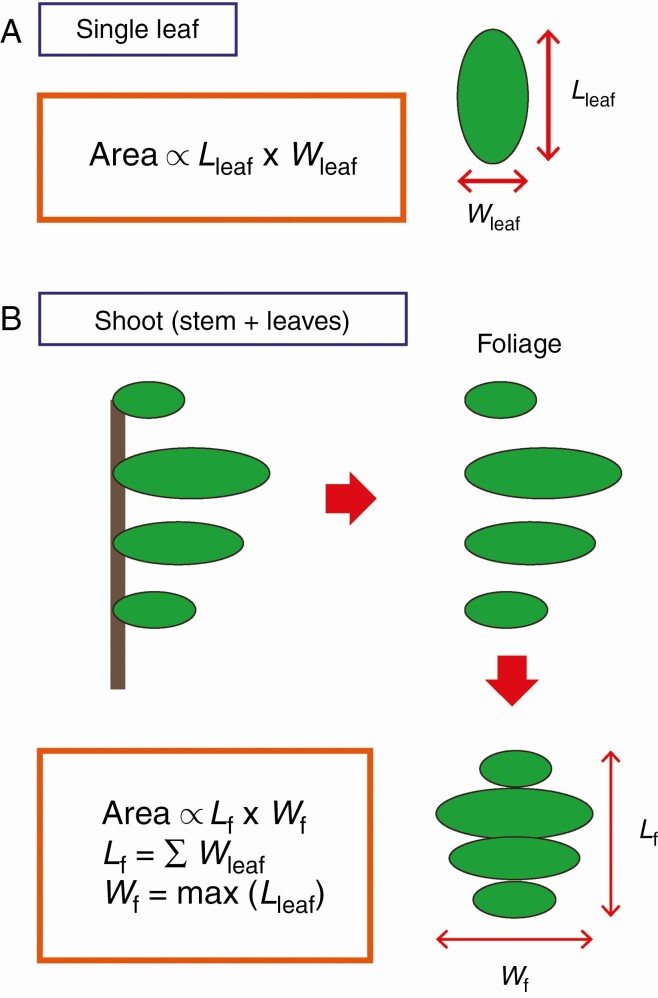
The length-times-width model for (A) an individual leaf and (B) a shoot.


First, we focused on individual leaves. Within a species, the area of an individual leaf (*A*_leaf_) is proportional to the lamina length (*L*_leaf_) times lamina width (*W*_leaf_) (Cain and Castro, 1959; Teobaldelli *et al*., 2019*a*, *b*; Yu *et al.*, 2020; Huang *et al.*, 2021; Li *et al.*, 2021; Schrader *et al.*, 2021; Shi *et al.*, 2021*a*) (Fig. 2A):

$$\begin{array}{l} A_{leaf}\propto L_{leaf}\times W_{leaf} \end{array}$$ (2)

The symbol ‘∝’ indicates ‘proportional to’. Equation (2) is known as the Montgomery equation (Yu *et al.*, 2020; Shi *et al.*, 2021*a*). It indicates that leaves from the same species are affine to each other.

Next, we extend eqn (2) to the level of shoots to predict *A*_shoot_. We hypothetically detach all the leaf laminas from the stem, and place them side-by-side on a flat plane to determine its dimensions as illustrated in Fig. 2B:


$$\begin{array}{l} \left\{ \begin{array}{ll} & L_{f}\equiv \sum_{shoot}W_{leaf}\\ & W_{f}\equiv \underset{shoot}{\max}\;\left(L_{leaf}\right) \end{array}\right. \end{array}$$ (3)

We refer to this set of leaf laminas as the ‘foliage’ of each shoot. The subscripts ‘f’ in eqn (3) stand for ‘foliage’. We exclude petioles because they contribute to the 3D arrangement with relatively little contribution to *A*_shoot_. The utility of rearranging the leaves is that both foliage length (*L*_f_) and foliage width (*W*_f_) can be defined independently from the 3D arrangement of the leaves. Our main hypothesis is that, within a single species, different-sized sets of foliage are affine, as is the case of individual leaves. This indicates that the total area of a foliage (which is *A*_shoot_, by definition) is proportional to the product of *L*_f_ and *W*_f_ (Fig. 2B):


$$\begin{array}{l} A_{shoot}\propto L_{f\;}\times \;W_{f} \end{array}$$ (4)

We will call eqn (4) the ‘foliage length-times-width equation’. Note that foliage *length* (*L*_f_) is defined using leaf *width* (not leaf length), and foliage width (*W*_f_) is defined using leaf *length*. The reason for these definitions is that both ‘leaf length’ and ‘foliage length’ are defined in the proximal–distal direction. In other words, foliage is analogous to a pinnately compound leaf that extends from the parent stem in the distal direction.

Next, we compare different-sized shoots from a single species. As shoot size increases, both *L*_f_ and *W*_f_ increase. We assume that the ratio of the relative growth rates of the foliage in these two directions is constant (Huxley, 1932; Niklas, 1994; Okie, 2013), and therefore follows the allometric relationship:


$$\begin{array}{l} L_{f}\propto W_{f}{}^{\beta }\;\left(\beta >1\right) \end{array}$$ (5)

The exponent *β* is expected to be >1, for the following reason. If foliage always consists of a single leaf, irrespective of its size, by definition *L*_f_ and *W*_f_ are equivalent to *W*_leaf_ and *L*_leaf_, respectively. In this case, *L*_f_ and *W*_f_ should be approximately proportional to each other (i.e. *β* ≈ 1). However, in reality, a shoot usually has multiple leaves. Because *L*_f_ is defined as the sum of the widths of *all* leaves, larger foliage with more leaves should have a disproportionately larger length relative to its width than small foliage (*β* > 1). In general, the value of *β* may vary among species, depending on the species’ intrinsic maximum leaf size and leafing intensity. In the Results, we show that eqn (5) is valid. Before demonstrating this, we proceed by assuming that eqn (5) is valid to derive other predictions. By combining eqns (4) and (5), we obtained:


$$\begin{array}{l} A_{shoot}\propto \;W_{f}{}^{\beta +1}\equiv {\left[\underset{shoot}{\max}\;\left(L_{leaf}\right)\right]}^{\beta +1} \end{array}$$ (6)

As mentioned above, the lamina area of an individual leaf is predicted by the product of lamina length and width with high accuracy (i.e. high *R*^2^ values). Additionally, it is known that individual leaf area can also be predicted by a quadratic function of lamina length or width alone [e.g. *A*_leaf_ ∝ (*L*_leaf_)^2^], albeit with less accuracy (Teobaldelli *et al*., 2019*a*, *b*). Similarly, eqn (6) predicts that *A*_shoot_ can also be predicted by *W*_f_ alone, with less but acceptable accuracy. Note that because *β* > 1, the exponent is expected to be >2. Suppose we further use an empirical relationship that individual lamina length is approximately proportional to lamina width [*L*_leaf_ ∝ *W*_leaf_ (Ogawa *et al.*, 1995)], by using eqn (6), we predicted that the maximum leaf lamina width within a shoot can also be used as a predictor of *A*_shoot_:


$$\begin{array}{l} A_{shoot}\propto {\left[\underset{shoot}{\max}\;\left(W_{leaf}\right)\right]}^{\beta +1} \end{array}$$ (7)

These predictions [eqns (6) and (7)] were also tested in this study. Previous studies have already recognized the usefulness of maximum leaf size as a predictor of *A*_shoot_ (Lopes and Pinto, 2005; Heerema *et al.*, 2008; Sun *et al.*, 2019*a*; Teobaldelli *et al.*, 2020). However, these studies used maximum leaf size only as empirical models. Therefore, none of them has provided a quantitative theory that explains why this relationship holds. In the following subsections, we show that these empirical relationships can also be derived as corollaries of the present model.

### Sun et al.’s equation

Sun *et al*. (2019*a*, 2020) found that *A*_shoot_ is proportional to the product of the maximum leaf area and total number of leaves on each shoot (*N*), because the maximum individual leaf area of a shoot corresponds to its potential leaf-producing capacity. This relationship can also be derived from our model (see Appendix for derivation):


$$\begin{array}{l} A_{shoot}\propto N\cdot \underset{shoot}{\max}\;\left(A_{leaf}\right) \end{array}$$ (8)

We retest this prediction in this study.

### Size–number allometry

We also derived the allometric relationship between *A*_shoot_ and the total number of leaves on each shoot (*N*) reported by Koyama *et al.* (2012) (see Appendix for derivation):


$$\begin{array}{l} \begin{array}{ll} & A_{shoot}\propto N^{\alpha },\\ & \;where\;\alpha \equiv \frac{\beta +1}{\beta -1}=1+\frac{2}{\beta -1}>1\;\left(∵\beta >1\right) \end{array} \end{array}$$ (9)

Generally, the exponent *α* may vary depending on species as a function of *β*. The predicted allometric relationship between *A*_shoot_ and *N* with the exponent *α* > 1 (given *β* > 1) agrees with the empirical result reported by Koyama *et al.* (2012). We retested this prediction in this study. In addition, eqn (9) can be rearranged to predict the scaling relationship between mean individual leaf area (=*A*_shoot_/*N*) and *A*_shoot_ with the exponent 0 < *λ* < 1 (see Appendix for derivation):


$$\begin{array}{l} \begin{array}{ll} & \frac{A_{shoot}}{N}\propto {\left(A_{shoot}\right)}^{\lambda },\\ & where\;\left\{ \begin{array}{ll} & \lambda \equiv 1-\frac{1}{\alpha }=\frac{2}{\beta +1}\\ & 0<\lambda <1\;\left(∵\beta >1\right) \end{array}\right. \end{array} \end{array}$$ (10)

The prediction that 0 < λ < 1 was empirically supported by Smith *et al.* (2017).

### Heerema–Spann–Teobaldelli et al.’s equation

Heerema, Spann and their colleagues (Heerema *et al.*, 2008; Spann and Heerema, 2010) reported an empirical relationship that *A*_shoot_ can be predicted by the maximum leaf length of a shoot (i.e. foliage width, *W*_f_) and the total number of leaves on that shoot (*N*) using woody fruit crop species. Teobaldelli *et al.* (2020) modified this relationship into a general allometric form. These relationships can also be derived from our model (see Appendix for derivation):


$$\begin{array}{l} \begin{array}{ll} & A_{shoot}\propto W_{f}\cdot N^{\gamma },\\ & where\;\left\{ \begin{array}{ll} & \gamma \equiv \frac{\beta }{\beta -1}=1+\frac{1}{\beta -1}\\ & \gamma >1\;\left(∵\beta >1\right) \end{array}\right. \end{array} \end{array}$$ (11)

Equation (11) was proposed as an empirical model by Teobaldelli *et al.* (2020), which includes the formula proposed by Heerema, Spann and their colleagues as a specific case when *γ* = 1, which does not take into consideration the *β*-dependency of *γ*. Generally, *γ* may vary among species as a function of *β*. Here, eqn (11) was tested by the following allometric relationship:


$$\begin{array}{l} \frac{A_{shoot}}{W_{f}}\propto N^{{}^{\gamma }} \end{array}$$ (12)

We also directly tested eqn (12). Unlike eqns (4) and (5), eqn (12) does not use foliage length (*L*_f_) as a variable, and therefore eqn (12) can be tested independently.

### Lopes–Pinto’s equation

Lopes and Pinto (2005) found an empirical formula that predicts *A*_shoot_ for a wine grape variety using the maximum and minimum leaf area within each shoot. They found that each shoot’s mean individual leaf area can be estimated as the mean of maximum and minimum leaf area within that shoot. This relationship can also be derived from our model (see Appendix for derivation):


$$\begin{array}{l} A_{shoot}=k\cdot N\left[\frac{\underset{shoot}{\min}\;\left(A_{leaf}\right)+\underset{shoot}{\max}\;\left(A_{leaf}\right)}{2}\;\right] \end{array}$$ (13)

The symbol *k* is a proportionality constant. Lopes, Pinto and colleagues (Lopes and Pinto, 2005; Phinopoulos *et al.*, 2015) found the same relationship as eqn (13) for two wine grape varieties as an empirical formula. They used an empirical value of *k* = 1 (i.e. in their cases, mean individual leaf area was simply the average value of the largest and the smallest leaves) as a specific value for the grape varieties. Generally, *k* may vary depending on the species (depending on the arrangement of different-sized leaves along a shoot). This prediction was also tested in this study.

## MATERIALS AND METHODS

### Study species

The study species and the sample sizes are listed in Table 1. Each species is referred to by its genus name after its first mention. All species have simple leaves with reticulate or reticulate-like venation patterns. (1) Kobushi magnolia (*Magnolia kobus*, Magnoliaceae). *Magnolia* was selected because it is taxonomically separate from the other species (APG IV, 2016). (2) *Cardiocrinum cordatum* (including var. *glehnii*) (Liliaceae) is a monocarpic perennial herb. This species belongs to the monocots (APG IV, 2016), but its leaves have reticulate venation patterns that are similar to those of eudicots (see photographs in Koyama *et al.*, 2012). Small individual plants form rosettes on the ground without elongating their stems, whereas large plants become bolting rosettes, which elongate their vertical stems with flower buds on top (Ohara *et al.*, 2006; Komamura *et al.*, 2021). (3) Sargent’s cherry (*Prunus sargentii*, Rosaceae) and (4) Japanese elm (*Ulmus davidiana* var. *japonica*, Ulmaceae). *Prunus* and *Ulmus* were chosen as typical broadleaved deciduous trees in temperate forests. (5) Giant knotweed (*Fallopia sachalinensis*, Polygonaceae) is a high-stature erect herb (plant height often reaches 2–3 m) with large leaves along its vertical stem. *Cardiocrinum* and *Fallopia* were chosen because they have contrasting growth forms (rosette vs. erect) and are from different taxonomic groups (monocot vs. eudicot).

**Table 1.** Study species and sample sizes

			*Magnolia kobus*	*Cardiocrinum cordatum*	*Prunus sargentii*	*Ulmus davidiana* var. *japonica*	*Fallopia sachalinensis*
Taxonomy			Magnoliid (Magnoliales, Magnoliaceae)	Monocot (Liliales, Liliaceae)	Eudicot (Rosales, Rosaceae)	Eudicot (Rosales, Ulmaceae)	Eudicot (Caryophyllales, Polygonaceae)
Growth form			Tree (deciduous)	Herb (rosette or bolting)	Tree (deciduous)	Tree (deciduous)	Erect herb
Location			R, T	F, H	U	U, F	U
Number of shoots investigated			37	36	39	43	29
Size ranges	*A*_shoot_ (cm^2^)	min	11.3	11.6	9.5	1.2	62.3
		max	1440.8	5718.2	1884.6	652.3	11 716.5
	*N*	min	2	1	1	1	4
		max	11	22	20	15	19

Location of sampling: F: The Forest of Obihiro; H: natural forest preservation of Hokkaido Obihiro Agricultural High School; R: Urikari River; T: Tokachi Ecology Park; U: Obihiro University of Agriculture and Veterinary Medicine. *A*_shoot_: total leaf area of each shoot (cm^2^); *N*: total leaf number of each shoot.

### Field sampling

A shoot is defined herein as a single current-year stem with its appendages (leaves, buds, flowers, fruits, etc.). For the two single-stemmed herbaceous species (*Cardiocrinum* and *Fallopia*), a shoot is equivalent to an entire above-ground part of an individual ramet, and therefore *A*_shoot_ is equivalent to whole-plant leaf area. Sample sizes and the sampling locations are given in Table 1. Sampling was conducted in summer (June–August) in 2016 and 2020. All sampling sites were located in Obihiro City or the adjacent Otofuke Town in Hokkaido Island in a cool-temperate region of Japan, and were within 10 km from the Obihiro Weather Station (42°52′N 143°10′E, altitude: 76 m a.s.l.). Mean annual temperature and precipitation at the weather station during 1998–2017 were 7.2 °C and 937 mm, respectively (Japan Meteorological Agency, 2020). Shoots with obvious damage (e.g. leaf loss due to herbivory etc.) were excluded. For the woody species, shoots that had sylleptic shoots (i.e. branching within the current year) were not sampled. Our sampling strategy was not random, but instead the shoots were sampled to cover a wide range sizes within each species (i.e. small, medium and large shoots were intentionally selected). Because healthy shoots were selected based solely on their sizes, both shaded and well-lit shoots were sampled for trees. For herbaceous species (*Cardiocrinum* and *Fallopia*), all shoots (ramets) within the same species grew in similar environments in their natural habitats. *Cardiocrinum* were sampled in partially shaded forest understories or small gaps and *Fallopia* were sampled in open clearings. For *Cardiocrinum*, leaf sizes were measured non-destructively *in situ* (see below). For the other species, shoots were harvested using pruning scissors or a long-reach pruner, sometimes with the aid of a stepladder. Immediately after sampling, shoots were stored in closed plastic bags with wet paper towels to avoid desiccation. Scanning (described below) was conducted within the same sampling day.

### Leaf size measurements

Leaf length (*L*_leaf_) is defined as the length of the leaf lamina, measured from the lamina tip to the point at which the lamina attaches to the petiole. Leaf width (*W*_leaf_) is defined as the maximum lamina width perpendicular to the midvein. Individual leaf area (*A*_leaf_) is defined as the area of one side of each lamina (John *et al.*, 2017). For *Cardiocrinum*, we measured *L*_leaf_ and *W*_leaf_ of all leaves on each stem using a measuring tape *in situ*. Then, *A*_leaf_ for this species was estimated using the following equation: individual leaf area = 0.7169 (leaf length × width) (Koyama *et al.*, 2012). For the other species, the harvested leaves were scanned using flatbed digital scanners (LiDE 210, Canon, Tokyo, Japan, 400 dpi; or 400-SCN025, Sanwa Supply, Okayama, Japan, 600 dpi). The sizes (*L*_leaf_, *W*_leaf_, *A*_leaf_) of each leaf were measured using ImageJ v.1.50i or 1.53a (Schneider *et al.*, 2012). For *Cardiocrinum*, both reproductive (large bolting plants) and vegetative shoots (rosettes) were sampled to cover the natural size range of this species, and the flower buds on top of *Cardiocrinum* stems were excluded as leaves. No reproductive organs were found among the sampled shoots of the other species. Some large shoots of *Prunus* and *Ulmus* trees, and most of the shoots of the erect herb *Fallopia*, were still elongating at the time of harvesting (June–August). For these shoots, only leaves of which laminae were unfolded (even when they were young and still expanding) were counted and measured; small folded immature leaves or leaf primordia near or at the shoot apical meristem were excluded as leaves. Among large shoots of *Fallopia*, small leaves were occasionally found on small lateral shoots that were branched from the main stem. These small lateral shoots were not measured because we focused on a single stem in this study. The total amount of those immature and lateral leaves was small compared to the total amount of leaves on the main stem.

All statistical analyses were performed with the statistical software R v.4.1.0 (R Core Team, 2021) and the packages *cowplot* (Wilke, 2016), *ggplot2* (Wickham, 2016), *gridExtra* (Auguie, 2017) and *smatr* (Warton *et al.*, 2012). Following Warton *et al.* (2006), ordinary least squares (OLS) and/or standardized major axis (SMA) regression analyses were performed for each relationship. OLS lines were fitted to predict variable *Y* (e.g. *A*_shoot_) from *X* (e.g. *L*_f_ × *W*_f_) with the R function *lm*. SMA lines were fitted to determine the mutual allometric relationship between two variables (e.g. foliage length vs. width) with the *sma* function of the package *smatr*. The *R*^2^ values of the OLS lines reported in this article were adjusted.

## RESULTS

**Fig. 3.**
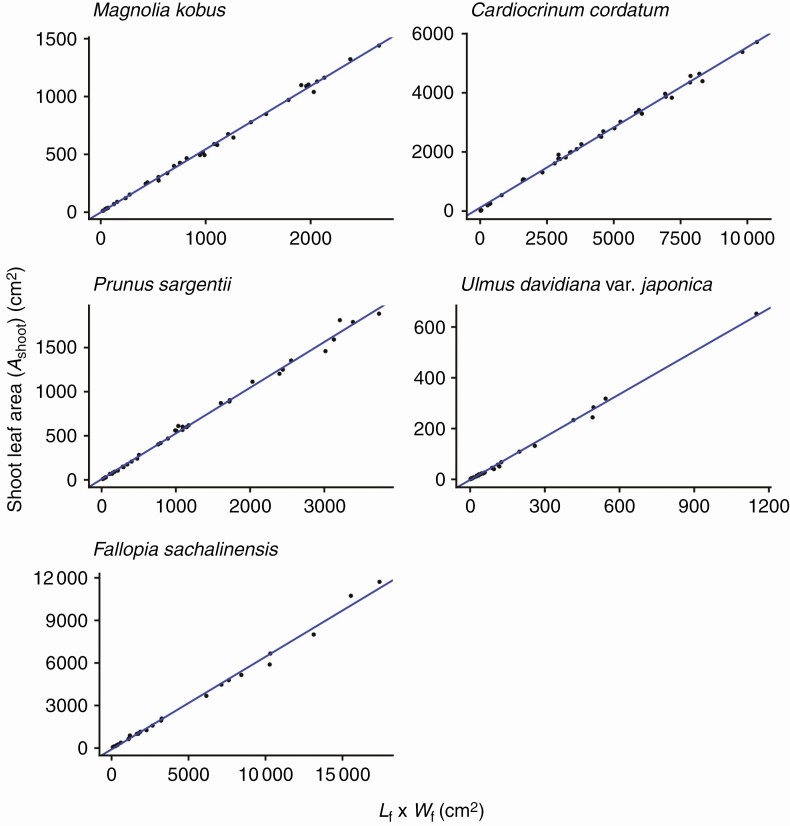
The total leaf area of a shoot (*A*_shoot_) is proportional to the product of foliage length (*L*_f_) and width (*W*_f_), as predicted by eqn (4). Each closed circle indicates an individual shoot. See Fig. 2B for the definition of foliage length and width. The blue lines show OLS (ordinary least squares) regression lines (*R*^2 ^> 0.994). See Table 2 for the regression results.

**Fig. 4.**
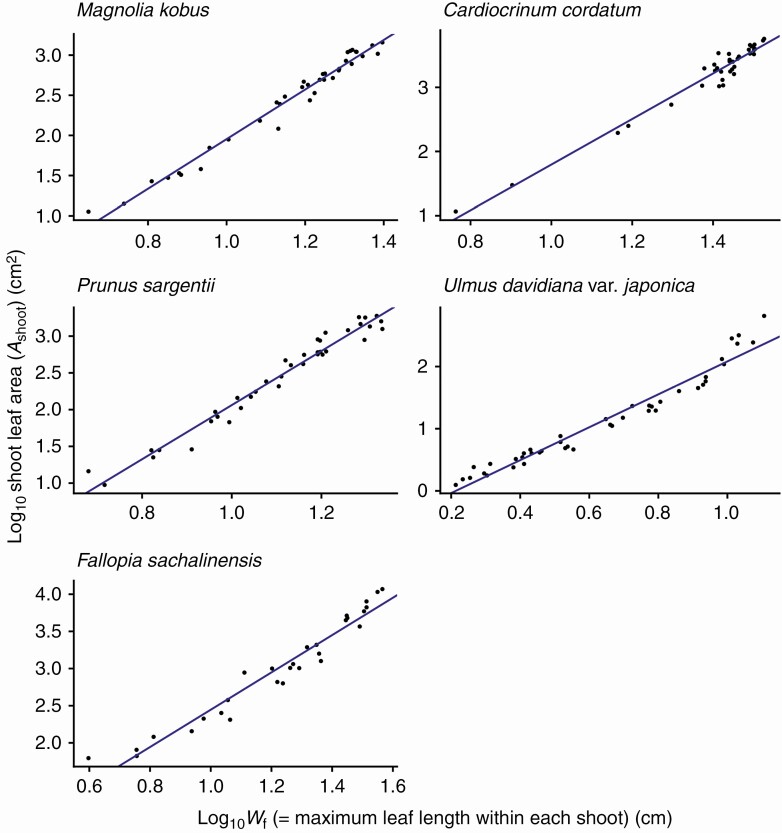
The total leaf area of a shoot (*A*_shoot_) is a power function of foliage width (*W*_f_), defined as the maximum individual leaf length of the shoot, as predicted by eqn (6). Each closed circle indicates one individual shoot. The blue lines show the OLS (ordinary least squares) regression lines (*R*^2 ^= 0.944–0.976). See Table 2 for the regression results.

**Fig. 5.**
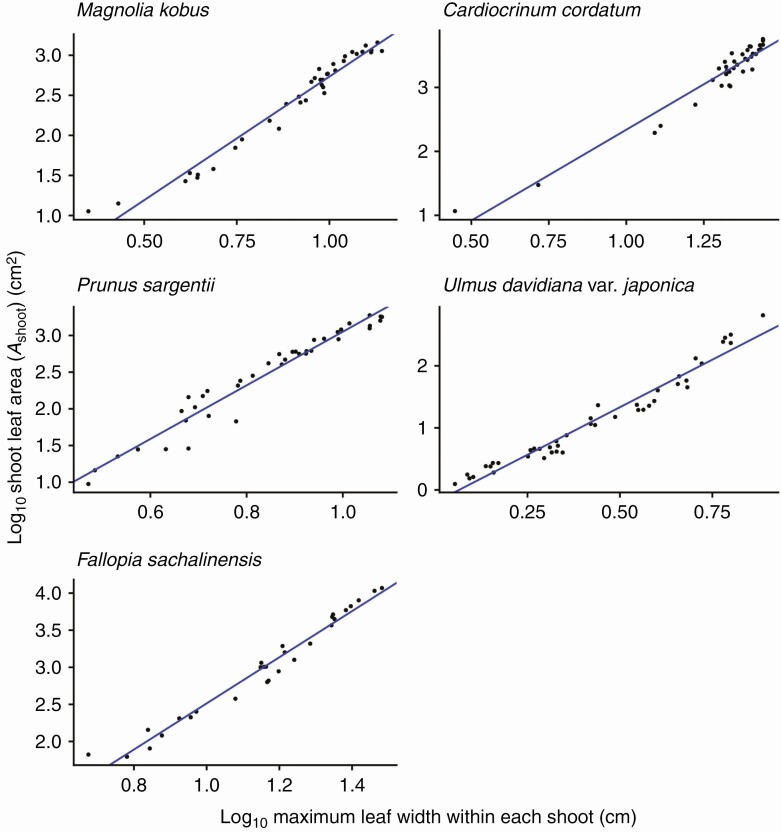
The total leaf area of a shoot (*A*_shoot_) is a power function of the maximum individual leaf width of the shoot, as predicted by eqn (7). Each closed circle indicates an individual shoot. The blue lines show the OLS (ordinary least squares) regression lines (*R*^2^ = 0.930–0.967). See Table 2 for the regression results.

**Fig. 6.**
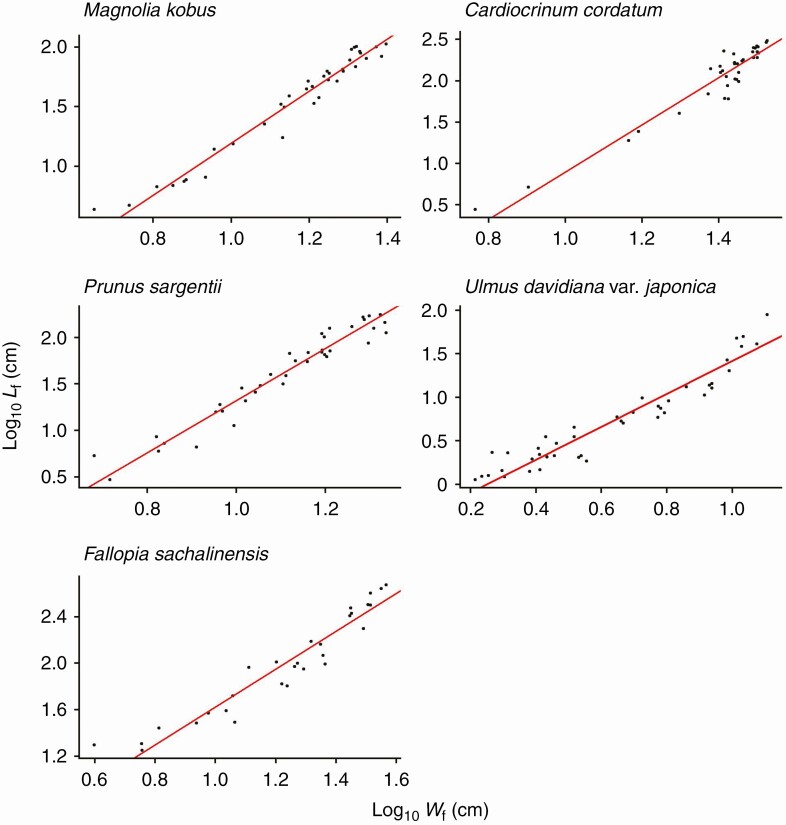
Log–log linear (allometric) relationship between foliage length (*L*_f_) and width (*W*_f_). The regression slopes correspond to *β* in eqn (5). Each closed circle indicates an individual shoot. The red lines show the SMA (standardized major axis) regression lines (*R*^2 ^= 0.905–0.960). See Table 2 for the regression results.

For all species investigated, the foliage length-times-width equation (eqn 4) explains *A*_shoot_ with high accuracy (*R*^2^ > 0.994 for all species; Fig. 3; Table 2). As predicted by Eqns (6) and (7), *A*_shoot_ can also be predicted as an allometric equation of maximum leaf length (i.e. foliage width *W*_f_; Fig. 4; Table 2) or maximum leaf width alone (Fig. 5; Table 2), though with less accuracy (OLS: *R*^2 ^= 0.930–0.976; SMA: 0.932–0.977). The allometric relationship between foliage width and length (eqn 5) was also supported (*R*^2 ^= 0.905–0.960; Fig. 6; Table 2). As expected, the scaling exponent *β* was >1, and the value of *β* varied greatly among the species (Table 2).

**Table 2.** Results of the regression analyses (OLS: ordinary least squares; SMA: standardized major axis). All regressions are significant (*P* < 1.0 × 10^ − 5^ for all cases).

*Y* = *a* + *bX*		Type		Mgk	Cac	Prs	Udj	Fas
*Y*	*X*							
$A_{shoot}$	$\begin{array}{ll} & L_{f\;}\times \;W_{f}\\ & \left\{ \begin{array}{ll} & L_{f}\equiv \sum_{shoot}W_{leaf}\\ & W_{f}\equiv \underset{shoot}{\max}\;\left(L_{leaf}\right) \end{array}\right. \end{array}$	OLS	*a*	−1.492	117.352	7.904	−2.109	−90.461
			*b*	0.546	0.543	0.519	0.562	0.652
			*R* ^2^	0.997	0.996	0.996	0.997	0.994
$\log_{10}\left(A_{shoot}\right)$	$\begin{array}{ll} & \log_{10}W_{f}\\ & =\log_{10}\left[\underset{shoot}{\max}\;\left(L_{leaf}\right)\right] \end{array}$	OLS	*a*	−1.135	−1.758	−1.620	−0.565	−0.065
			*b*	3.088	3.554	3.682	2.647	2.511
			*R* ^2^	0.976	0.957	0.966	0.951	0.944
		SMA	*a*	−1.178	−1.866	−1.690	−0.606	−0.151
			*b*	3.125	3.630	3.745	2.712	2.582
			*R* ^2^	0.977	0.958	0.967	0.953	0.946
$\log_{10}\left(A_{shoot}\right)$	$\log_{10}\left[\underset{shoot}{\max}\;\left(W_{leaf}\right)\right]$	OLS	*a*	−0.350	−0.502	−0.603	−0.199	−0.598
			*b*	3.084	2.841	3.654	3.064	3.111
			*R* ^2^	0.963	0.930	0.946	0.956	0.967
		SMA	*a*	−0.402	−0.635	−0.687	−0.228	−0.657
			*b*	3.141	2.943	3.754	3.132	3.162
			*R* ^2^	0.964	0.932	0.947	0.957	0.968
$\log_{10}L_{f}$	$\log_{10}W_{f}$	SMA	*a*	−0.939	−1.783	−1.387	−0.415	0.090
			*b* (=*β*)	2.187	2.856	2.806	1.892	1.629
			*R* ^2^	0.961	0.916	0.939	0.907	0.909
$\log_{10}\left(A_{shoot}\right)$	$\log_{10}N$	OLS	*a*	0.742	1.853	0.830	0.333	0.103
			*b*	2.325	1.443	1.865	1.980	3.137
			*R* ^2^	0.963	0.857	0.946	0.855	0.922
		SMA	*a*	0.711	1.747	0.785	0.271	−0.013
			*b* (=*α*)	2.368	1.555	1.916	2.136	3.263
			*R* ^2^	0.964	0.861	0.948	0.859	0.925
$A_{shoot}$	$N\cdot \underset{shoot}{\max}\;\left(A_{leaf}\right)$	OLS	*a*	24.144	420.913	33.972	−4.077	−97.621
			*b*	0.630	0.369	0.637	0.690	0.712
			*R* ^2^	0.980	0.976	0.987	0.996	0.989
$\log_{10}\left(\frac{A_{shoot}}{W_{f}}\right)$	$\log_{10}N$	OLS	*a*	0.111	0.796	0.137	−0.029	−0.065
			*b*	1.605	1.080	1.390	1.288	1.997
			*R* ^2^	0.977	0.896	0.962	0.871	0.943
		SMA	*a*	0.097	0.740	0.114	−0.064	−0.118
			*b* (=*γ*)	1.623	1.139	1.416	1.378	2.054
			*R* ^2^	0.977	0.899	0.963	0.874	0.945
$A_{shoot}$	$N\left[\frac{\underset{shoot}{\min}\;\left(A_{leaf}\right)+\underset{shoot}{\max}\;\left(A_{leaf}\right)}{2}\right]$	OLS	*a*	7.376	310.017	−16.200	−5.339	−122.219
			*b* (=*k*)	1.092	0.758	1.109	1.193	1.221
			*R* ^2^	0.994	0.981	0.991	0.992	0.993
$A_{leaf}$	$L_{leaf}\cdot W_{leaf}$	OLS	*a*	1.163	–	−0.799	0.121	1.514
			*b*	0.648	–	0.643	0.663	0.798
			*R* ^2^	0.989	–	0.992	0.992	0.992

Mgk: *Magnolia kobus*; Cac: *Cardiocrinum cordatum*; Prs: *Prunus sargentii*; Udj: *Ulmus davidiana* var. *japonica*; Fas: *Fallopia sachalinensis*.

**Fig. 7.**
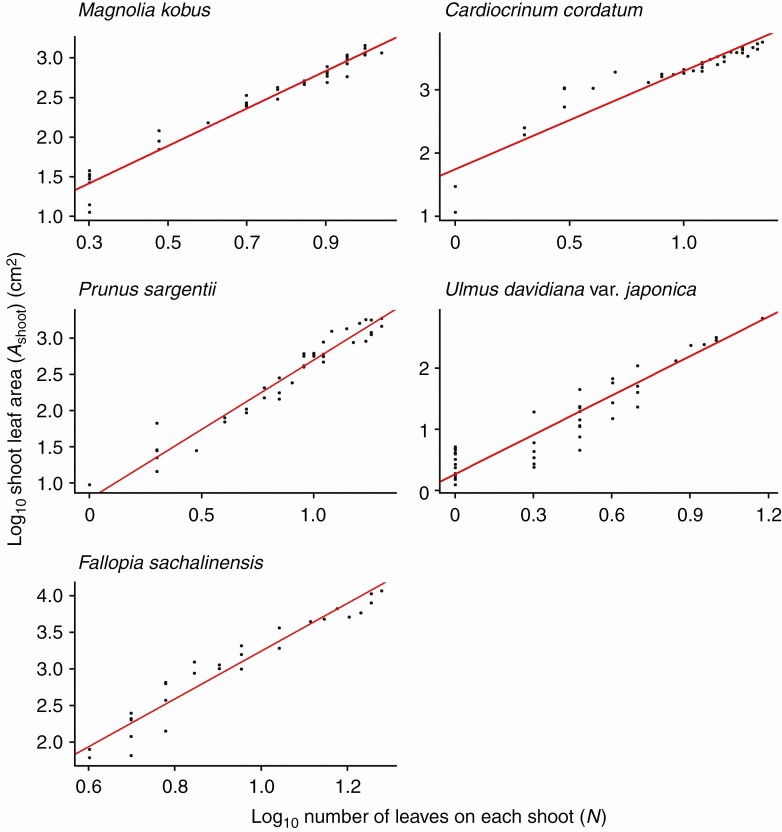
Log–log linear (allometric) relationship between the total leaf area of a shoot (*A*_shoot_) and the total number of leaves on the shoot (*N*). The regression slopes correspond to *α* in eqn (9). Each closed circle indicates one individual shoot. The red lines show the SMA (standardized major axis) regression lines (*R*^2 ^= 0.859–0.964). See Table 2 for the regression results.

**Fig. 8.**
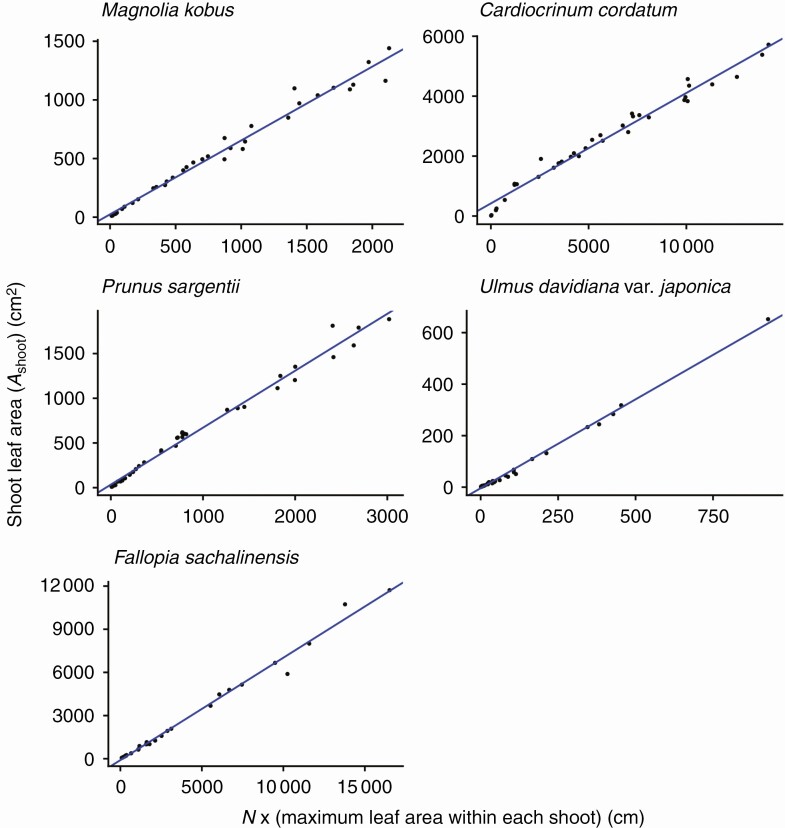
The total leaf area of a shoot (*A*_shoot_) is proportional to the product of the maximum individual leaf area and the number of leaves on the shoot (*N*), as predicted by eqn (8). Each closed circle indicates one individual shoot. The blue lines show the OLS (ordinary least squares) regression lines (*R*^2 ^> 0.976).

**Fig. 9.**
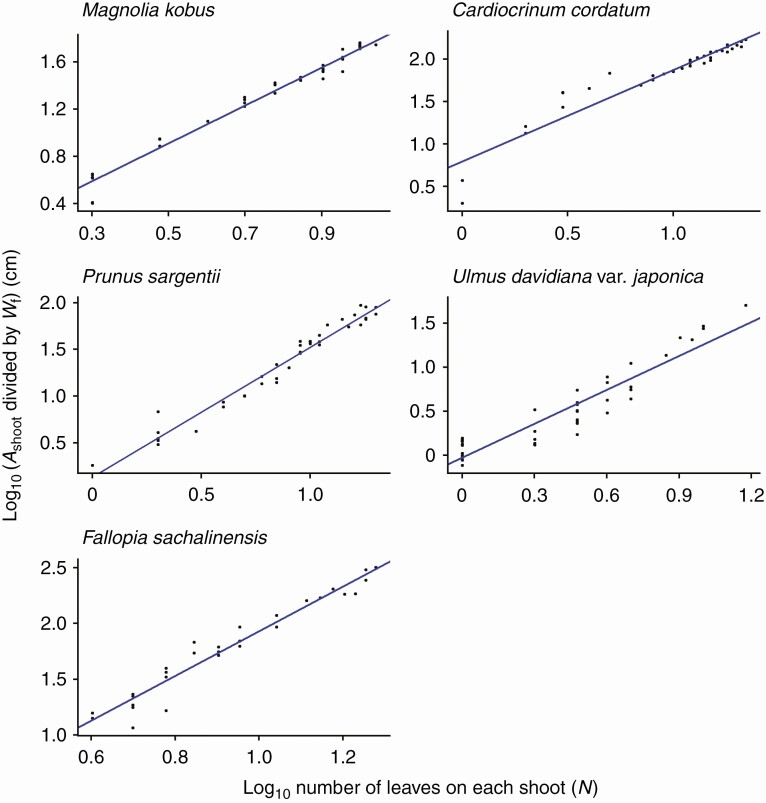
The total leaf area of a shoot divided by foliage width (*A*_shoot_/*W*_f_) is a power function of the number of leaves on the shoot (*N*). The regression slopes correspond to *γ* in eqn (12). Each closed circle indicates an individual shoot. The blue lines show the OLS (ordinary least squares) regression lines (*R*^2 ^= 0.871–0.977). See Table 2 for the regression results.

**Fig. 10.**
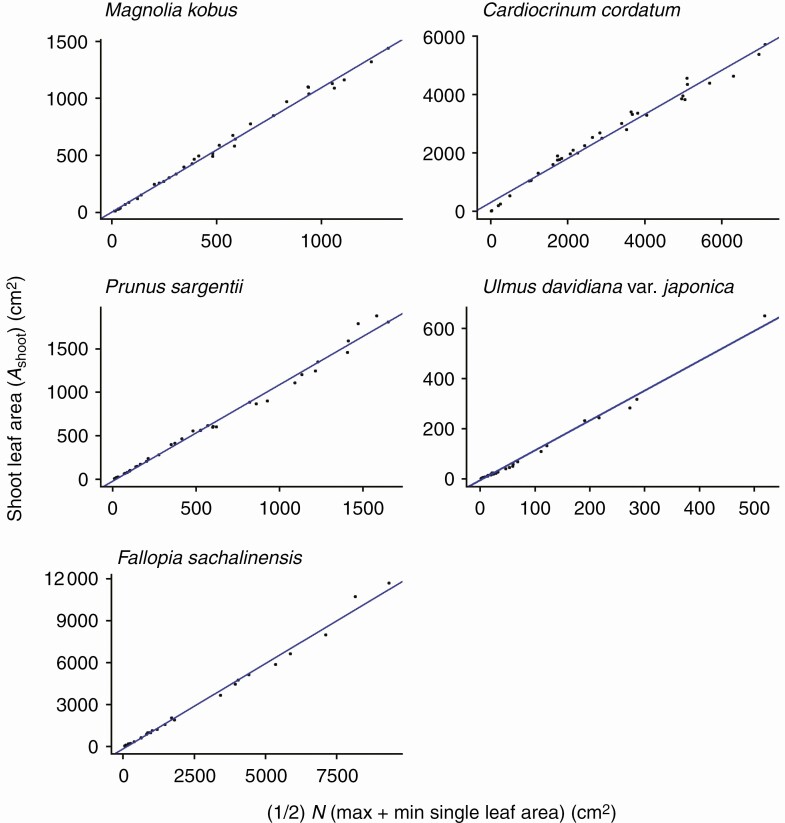
The total leaf area of a shoot (*A*_shoot_) is proportional to the product of the number of leaves (*N*) times (maximum + minimum individual leaf area) divided by 2. The regression slopes correspond to *k* in eqn (13). Each closed circle indicates an individual shoot. The blue lines show the OLS (ordinary least squares) regression lines (*R*^2 ^> 0.981). See Table 2 for the regression results.

As predicted by eqn (9), *A*_shoot_ is expressed as a power function of the total number of leaves on that shoot (*N*) with the SMA regression exponents >1 (Fig. 7; Table 2), though for this relationship substantial deviations from the regression lines (*R*^2^ = 0.859–0.964; Table 2) were observed in the region for *N* ≤ 3 (log_10_*N* ≤ 0.48). This is especially evident when *N* = 1, in which case *A*_shoot_ is represented by only a single leaf, and as *N* increases, the values of *A*_shoot_ become stable as they are calculated as the sum of many leaves. The present data also reconfirm all previously known empirical relationships found by Sun *et al.* [eqn (8); *R*^2^ > 0.976; Fig. 8; Table 2], by Heerema–Spann–Teobaldelli *et al*. [eqn (12); OLS: *R*^2^ = 0.871–0.977; SMA: *R*^2^ = 0.874–0.977; Fig. 9; Table 2] and by Lopes–Pinto [eqn (13); *R*^2^ > 0.981; Fig. 10; Table 2]. The present data also reconfirm the leaf-level relationships that individual leaf area is proportional to the product of its lamina length and width [eqn (2); *R*^2^ > 0.989; Table 2].

## Discussion

### Leaf vs. shoot elongation

The structure of a shoot, including size variation and arrangement of leaves, determines the light-harvesting efficiency of plants (Givnish, 1984; Valladares and Brites, 2004; Pearcy *et al.*, 2005; Smith *et al.*, 2017; Olson *et al.*, 2018; Koyama *et al.*, 2020; Iwabe *et al.*, 2021). If a shoot is to minimize the cost of current light harvesting, the optimal solution derived by Givnish (1982) is to have a single large leaf with no investment in the stem (i.e. no stem elongation). Why does a shoot have multiple leaves instead of a single large leaf? There are mutually non-exclusive explanations for the benefit of producing multi-leaved stems rather than single-leaved stems. First, plants are subject to competition with neighbours (Givnish, 1982; Anten, 2016), and existing leaves will be gradually shaded by neighbouring plants in the future. Under competition, plants should continuously elongate their stems and produce new leaves in better-lit positions (Koyama and Kikuzawa, 2009; Anten, 2016; Koyama *et al.*, 2020). Therefore, a shoot has at least two functions in terms of light capture: current light harvesting and space acquisition, the latter of which contributes to future light harvesting (Yagi and Kikuzawa, 1999; Sterck *et al.*, 2005; Laurans and Vincent, 2016; Koyama *et al.*, 2020). Differentiation of short vs. long shoots can be considered as a continuum of a strategy along the trade-off between these two functions (Yagi and Kikuzawa, 1999). In the present dataset, the exponent *β* was >1 [eqn (5); Table 2], indicating that larger foliage had a larger foliage length relative to its foliage width, as expected because larger foliage consists of more leaves than smaller foliage (Fig. 7). This phenomenon is called geometric dissimilitude and it can be considered as a shift in strategy along size variation (Niklas, 1994; Okie, 2013). These results are consistent with the observation that long shoots are specialized for space acquisition whereas short shoots are specialized for light capture, and there is a continuous shift between these two extremes (Yagi and Kikuzawa, 1999). Second, larger leaves produce a thicker boundary layer that reduces heat and gas exchange (Schuepp, 1993; Xu *et al.*, 2009); therefore, larger leaves are subject to greater heat stress (Vogel, 2009). Having compound leaves that consist of multiple leaflets instead of simple large leaves can effectively reduce the boundary layer resistance (Gurevitch and Schuepp, 1990; Xu *et al.*, 2009). At the level of individual leaves, Schrader *et al.* (2021) demonstrated that the length-times-width equation (eqn 2) is valid for compound leaves. However, at the shoot level, leaf shape (e.g. simple vs. compound) may also affect the leaf–shoot allometric relationship (Yang *et al.*, 2009). Therefore, the scaling relationships may also be affected by the leaf shape, which is in turn is affected by the environment (Royer *et al.*, 2005; Xu *et al.*, 2009). Third, Kleiman and Aarssen (2007) suggested that producing more leaves, instead of fewer but larger ones, is more beneficial because it allows stems to have more buds and eventually leads to greater lateral growth and higher plasticity of allocation between growth and reproduction. Fourth, for a given limit on the total leaf area of a shoot, larger leaves incur a disproportionately greater cost of supporting tissues (Niinemets *et al.*, 2007; Shi *et al.*, 2020). Fifth, if a plant has many leaves, then the feeding or attacking efficiency of herbivores or pathogens may be reduced (Brown *et al.*, 1991). Altogether, the observed variation of *β* across the five species may reflect these multiple compounding factors. Therefore, further investigations on species with different leaf shapes (such as compound leaves), leaf sizes, leafing intensities and environments (including herbivores and pathogens) are needed.

In this study, we intentionally ignored the 3D arrangement of foliage and instead considered the 2D structure of foliage as being analogous to a single large leaf (Fig. 2B). By doing so, our length-times-width equation successfully predicted *A*_shoot_ with high accuracy without considering any details of the actual foliage structure other than size. The simplification applied in this study is in contrast to existing models, which consider the 3D arrangement of leaves, such as phyllotaxis (Valladares and Brites, 2004; Smith *et al.*, 2017), internode length (Meng *et al.*, 2013), stem inclination angle (Meng *et al.*, 2013), and the resultant light interception and within-shoot self-shading (Valladares and Brites, 2004; Koyama and Kikuzawa, 2010; Smith *et al.*, 2017; Olson *et al.*, 2018). Our model does not consider stem traits, such as cross-sectional area (Brouat *et al.*, 1998; Yan *et al.*, 2013; Smith *et al.*, 2017; Lehnebach *et al.*, 2018; Sun *et al*., 2019*a*, *b*, 2020; Fajardo *et al.*, 2020), length-to-diameter ratio (Xiang *et al.*, 2009*a*, Levionnois *et al.*, 2021), conduit size, which determines hydraulic efficiency (Savage *et al.*, 2010; Chen *et al.*, 2012; Fan *et al.*, 2017; Trueba *et al.*, 2019; Olson *et al.*, 2020; Bortolami *et al.*, 2021; Levionnois *et al.*, 2021), stem mechanical properties (Brouat and McKey, 2001; Chen *et al.*, 2009; Trueba *et al.*, 2016; Fan *et al.*, 2017; Olson *et al.*, 2018; Baer *et al.*, 2021; Levionnois *et al.*, 2021) or the associated stem construction costs (Yang *et al.*, 2010; Givnish, 2020). Nonetheless, because our model focuses only on a population of leaf laminas, it is not mutually exclusive to the previous models. Instead, the geometric property of foliage can be incorporated to improve the previous models, which do not consider the within-shoot size variation of individual leaves.

### Limitations of the model

The product of leaf lamina length and width can predict individual leaf area, and this relationship holds for diverse taxa and for different growth conditions, without considering the underlying leaf structures such as venation (Blonder *et al.*, 2020; Kawai and Okada, 2020), lobation (Schuepp, 1993; Kusi and Karsai, 2020), lamina folding (Fleck *et al.*, 2003; Deguchi and Koyama, 2020), epidermal features (Maslova *et al.*, 2021) and internal mesophyll structures (Oguchi *et al.*, 2005), all of which are known to differ among angiosperm species and under different environmental conditions. The consistency of the shoot-level results among eudicots, Magnoliids and monocots obtained herein may imply that the present results may also be generalized across angiosperms, as is the case for individual leaves. However, because our aim was to propose and test a new model as a starting point, we chose only five typical temperate woody and herbaceous species. In general, leaf–shoot allometric relationships are affected by climate or altitude (Westoby and Wright, 2003; Sun *et al*., 2006, 2019*a*; Xiang *et al.*, 2009*a*, *b*, 2010; Zhu *et al.*, 2019), as well as by leaf habit (i.e. deciduous vs. evergreen) (Brouat *et al.*, 1998; Yang *et al.*, 2008, 2009; Milla, 2009; Zhu *et al.*, 2019; Fajardo *et al.*, 2020). Therefore, it remains unclear whether the present results can be applied to different situations, including other species from extreme climates or different life forms, such as evergreen conifers. Additionally, our model does not consider compound leaves. At the level of individual leaves, a recent study demonstrated that the length-times-width equation (eqn 2) is valid for both simple and compound leaves (Schrader *et al.*, 2021). However, at the level of shoots, leaf shape may also affect the leaf–shoot allometric relationship (Yang *et al.*, 2009). Therefore, more comprehensive datasets that include a diversity of leaf forms are needed to validate our model. Furthermore, our model does not consider the reproductive organs. The scaling relationships between reproductive organs and shoot size has long been recognized (Chen *et al.*, 2009; Scott and Aarssen, 2013; Miranda *et al.*, 2019), and the existence of reproductive organs also alters scaling relationship among vegetative organs (Fajardo *et al.*, 2020). Therefore, future studies are needed to elucidate whether the simple relationship found in the present study is affected by the existence of reproductive organs.

## Conclusions

Based on the geometric properties of foliage, we proposed the ‘foliage length-times-width equation’ that accurately predicts the total leaf area of a shoot. The model unifies several previously established empirical relationships into a single theory. We also demonstrated that the total leaf area of a shoot can also be predicted by maximum individual leaf lamina length or width alone. The dataset of five species from diverse taxa generally supported the model predictions, though deviations from the model were also observed. More comprehensive datasets that include a diversity of species are needed to test the generality of our model in future studies.

## Appendix

### Derivation of eqn (8)

When different-sized foliage sets are affine to each other, they become congruent (i.e. identical in shape) when normalized to the same size. This indicates that the maximum leaf length (or width) within each shoot relative to the mean value of the same shoot is constant, leading to the following relationships:

$$\begin{array}{l} \left\{ \begin{array}{ll} & \underset{shoot}{mean}\;\left(L_{leaf}\right)\propto \underset{shoot}{\max}\;\left(L_{leaf}\right)\\ & \underset{shoot}{mean}\;\left(W_{leaf}\right)\propto \underset{shoot}{\max}\;\left(W_{leaf}\right) \end{array}\right. \end{array}$$
(14)

By taking the product of both sides of the two lines in eqn (14), and using eqn (2), we obtained:

$$\begin{array}{l} \underset{shoot}{mean}\;\left(A_{leaf}\right)\propto \underset{shoot}{\max}\;\left(A_{leaf}\right) \end{array}$$
(15)

In deriving eqn (15), we assumed that the leaf that has maximum (or mean) length within each shoot also has the maximum (or mean) width in the same shoot. By multiplying both sides of eqn (15) by the total number of leaves on each shoot (*N*), we obtained:

$$\begin{array}{l} N\cdot \underset{shoot}{mean}\;\left(A_{leaf}\right)\propto N\cdot \underset{shoot}{\max}\;\left(A_{leaf}\right) \end{array}$$
(16)

Because the left-hand-side of eqn (16) is *A*_shoot_, we obtained eqn (8).

#### Derivation of eqn (9)

In this section, we assume that lamina length and width of an individual leaf are approximately proportional to each other (Ogawa *et al.*, 1995), and that individual leaf area can be predicted as the quadratic function of either leaf length or width alone (Teobaldelli *et al*., 2019a, b):

$$\begin{array}{l} A_{leaf}\propto {\left(L_{leaf}\right)}^{2} \end{array}$$
(17)

Equation (17) can also be used to predict the maximum *A*_leaf_ within each shoot:

$$\begin{array}{l} \underset{shoot}{\max}\;\left(A_{leaf}\right)\propto {\left[\underset{shoot}{\max}\;\left(L_{leaf}\right)\right]}^{2} \end{array}$$
(18)

By multiplying both sides of eqn (18) by the total number of leaves on each shoot (*N*), we obtained:

$$\begin{array}{l} N\cdot \underset{shoot}{\max}\;\left(A_{leaf}\right)\propto N\cdot {\left[\underset{shoot}{\max}\;\left(L_{leaf}\right)\right]}^{2} \end{array}$$
(19)

By combining eqns (8) and (19), we obtained:

$$\begin{array}{l} A_{shoot}\propto N\cdot {\left[\underset{shoot}{\max}\;\left(L_{leaf}\right)\right]}^{2} \end{array}$$
(20)

By using our definition of *W*_f_ (eqn 3), eqn (20) can be rewritten as follows:

$$\begin{array}{l} A_{shoot}\propto N\cdot W{{}_{f}}^{2} \end{array}$$
(21)

By combining eqns (6) and (21), we obtained:

$$\begin{array}{l} W{{}_{f}}^{\beta +1}\propto N\cdot W{{}_{f}}^{2} \end{array}$$
(22)

Equation (22) can be solved for *W*_f_:

$$\begin{array}{l} W{}_{f}\propto N^{\frac{1}{\beta -1}} \end{array}$$
(23)

By substituting eqn (23) into eqn (6), we obtained:

$$\begin{array}{l} A_{shoot}\propto N^{\frac{\beta +1}{\beta -1}}\equiv N^{\alpha } \end{array}$$
(24)

#### Derivation of eqn (10)

Equation (9) can be solved for *N*_f_:

$$N\alpha A_{\text{shoot}}{}^{\frac{1}{\alpha }}$$
(25)

Using eqn (25), we obtained:

$$\begin{array}{l} \frac{A_{shoot}}{N}\propto {\left(A_{shoot}\right)}^{1-\frac{1}{\alpha }}\equiv {\left(A_{shoot}\right)}^{\lambda } \end{array}$$
(26)

#### Derivation of eqn (11)

By combining eqns (5) and (23), we obtained:

$$L_{\text{f}}\alpha W_{\text{f}}^{\beta }\alpha N^{\frac{\beta }{\beta -1}}$$
(27)

By substituting eqn (27) into eqn (4), we obtained:

$$A_{\text{shoot}}\alpha W_{\text{f}}\cdot N^{\frac{\beta }{\beta -1}}\equiv W_{\text{f}}\cdot N^{\gamma }$$
(28)

#### Derivation of eqn (13)

When different-sized foliage sets are affine to each other, the maximum leaf size relative to the mean value and the minimum leaf size relative to the mean value would be constants, independent of foliage size. This leads to the following relationship:

$$\begin{array}{l} \underset{shoot}{mean}\;\left(A_{leaf}\right)\propto \underset{shoot}{\min}\;\left(A_{leaf}\right)\; \end{array}$$
(29)

By dividing both sides of eqns (15) and (29) by 2, and adding them, we obtained:

$$\begin{array}{l} \underset{shoot}{mean}\;\left(A_{leaf}\right)\propto \frac{\underset{shoot}{\min}\;\left(A_{leaf}\right)+\underset{shoot}{\max}\;\left(A_{leaf}\right)}{2} \end{array}$$
(30)

By multiplying both sides of eqn (30) by *N*, we obtained eqn (13).

## References

[CIT0001] Ackerly DD , Bazzaz FA. 1995. Leaf dynamics, self-shading and carbon gain in seedlings of a tropical pioneer tree. Oecologia 101: 289–298. doi:10.1007/BF00328814. 28307049

[CIT0002] Ackerly DD , Donoghue MJ. 1998. Leaf size, sapling allometry, and Corner’s rules: phylogeny and correlated evolution in maples (*Acer*). The American Naturalist 152: 767–791. doi:10.1086/286208. 18811427

[CIT0003] Anten NPR . 2016. Optimizationand game theory in canopy models. In: Hikosaka K, Niinemets Ü, Anten NPR, eds. Canopy photosynthesis: from basics to applications. Dordrecht: Springer Netherlands, 355–377.

[CIT0004] APG IV. 2016. An update of the Angiosperm Phylogeny Group classification for the orders and families of flowering plants: APG IV. Botanical Journal of the Linnean Society 181: 1–20.

[CIT0005] Auguie B . 2017. https://CRAN.R-project.org/package=gridExtra.

[CIT0006] Baer AB , Fickle JC, Medina J, Robles C, Pratt RB, Jacobsen AL. 2021. Xylem biomechanics, water storage, and density within roots and shoots of an angiosperm tree species. Journal of Experimental Botany 72: 7984–7997. 34410349 10.1093/jxb/erab384

[CIT0007] Banavar JR , Cooke TJ, Rinaldo A, Maritan A. 2014. Form, function, and evolution of living organisms. Proceedings of the National Academy of Sciences of the United States of America 111: 3332–3337. doi:10.1073/pnas.1401336111. 24550479 PMC3948298

[CIT0008] Bazzaz FA , Harper JL. 1977. Demographic analysis of the growth of *Linum usitatissimum*. New Phytologist 78: 193–208. doi:10.1111/j.1469-8137.1977.tb01558.x.

[CIT0009] Bentley LP , Stegen JC, Savage VM, et al 2013. An empirical assessment of tree branching networks and implications for plant allometric scaling models. Ecology Letters 16: 1069–1078. doi:10.1111/ele.12127. 23800188

[CIT0010] Blonder B , Both S, Jodra M, et al 2020. Linking functional traits to multiscale statistics of leaf venation networks. New Phytologist 228: 1796–1810. 32712991 10.1111/nph.16830

[CIT0011] Bortolami G , Farolfi E, Badel E, et al 2021. Seasonal and long-term consequences of esca grapevine disease on stem xylem integrity. Journal of Experimental Botany 72: 3914–3928. 33718947 10.1093/jxb/erab117

[CIT0012] Brouat C , Gibernau M, Amsellem L, McKey D. 1998. Corner’s rules revisited: ontogenetic and interspecific patterns in leaf–stem allometry. New Phytologist 139: 459–470.

[CIT0013] Brouat C , McKey D. 2001. Leaf-stem allometry, hollow stems, and the evolution of caulinary domatia in myrmecophytes. New Phytologist 151: 391–406.

[CIT0014] Brown VK , Lawton JH, Grubb PJ, Chaloner WG, Harper JL, Lawton JH. 1991. Herbivory and the evolution of leaf size and shape. Philosophical Transactions of the Royal Society of London, Series B: Biological Sciences 333: 265–272.

[CIT0015] Bultynck L , Ter Steege MW, Schortemeyer M, Poot P, Lambers H. 2004. From individual leaf elongation to whole shoot leaf area expansion: a comparison of three *Aegilops* and two *Triticum* species. Annals of Botany 94: 99–108. 15155374 10.1093/aob/mch110 PMC4242366

[CIT0016] Cain SA , Castro GDO. 1959. Manual of vegetation analysis. New York: Harper & Brothers.

[CIT0017] Chen H , Niklas KJ, Sun S. 2012. Testing the packing rule across the twig–petiole interface of temperate woody species. Trees 26: 1737–1745.

[CIT0018] Chen H , Niklas KJ, Yang D, Sun S. 2009. The effect of twig architecture and seed number on seed size variation in subtropical woody species. New Phytologist 183: 1212–1221. 19496950 10.1111/j.1469-8137.2009.02878.x

[CIT0019] Corner EJH . 1949. The durian theory or the origin of the modern tree. Annals of Botany 13: 367–414.

[CIT0020] Deguchi R , Koyama K. 2020. Photosynthetic and morphological acclimation to high and low light environments in *Petasites japonicus* subsp. *giganteus*. Forests 11: 1365.

[CIT0021] DeJong TM , Day KR, Johnson RS. 1989. Partitioning of leaf nitrogen with respect to within canopy light exposure and nitrogen availability in peach (*Prunus persica*). Trees-Structure and Function 3: 89–95.

[CIT0022] Dombroskie SL , Aarssen LW. 2012. The leaf size/number trade-off within species and within plants for woody angiosperms. Plant Ecology and Evolution 145: 38–45.

[CIT0023] Dombroskie SL , Tracey AJ, Aarssen LW. 2016. Leafing intensity and the fruit size/number trade-off in woody angiosperms. Journal of Ecology 104: 1759–1767.

[CIT0024] Enquist BJ , West GB, Brown JH. 2009. Extensions and evaluations of a general quantitative theory of forest structure and dynamics. Proceedings of the National Academy of Sciences of the United States of America 106: 7046–7051. 19363161 10.1073/pnas.0812303106 PMC2678479

[CIT0025] Fajardo A , Mora JP, Robert E. 2020. Corner’s rules pass the test of time: little effect of phenology on leaf–shoot and other scaling relationships. Annals of Botany 126: 1129–1139. 32598449 10.1093/aob/mcaa124 PMC7684704

[CIT0026] Falconer K . 2003. Fractal geometry: mathematical foundations and applications, 2nd edn. Chichester: John Wiley & Sons.

[CIT0027] Fan Z-X , Sterck F, Zhang S-B, Fu P-L, Hao G-Y. 2017. Tradeoff between stem hydraulic efficiency and mechanical strength affects leaf–stem allometry in 28 *Ficus* tree species. Frontiers in Plant Science 8: 1619. 10.3389/fpls.2017.01619 PMC5611361 28979282

[CIT0028] Field C . 1983. Allocating leaf nitrogen for the maximization of carbon gain - leaf age as a control on the allocation program. Oecologia 56: 341–347. 28310214 10.1007/BF00379710

[CIT0029] Field CB . 1991. Ecological scaling of carbon gain to stress and resource availability. In: Mooney HA, Winner WE, Pell EJ, eds. Response of plants to multiple stresses. San Diego: Academic Press, 35–65.

[CIT0030] Fleck S , Niinemets U, Cescatti A, Tenhunen JD. 2003. Three-dimensional lamina architecture alters light-harvesting efficiency in *Fagus*: a leaf-scale analysis. Tree Physiology 23: 577–589. 12750051 10.1093/treephys/23.9.577

[CIT0031] Givnish T . 1984. Leaf and canopy adaptations in tropical forests. In: Medina E, Mooney HA, Vázquez-Yánes C, eds. Physiological ecology of plants of the wet tropics. Dordrecht: Springer, 51–84.

[CIT0032] Givnish TJ . 1982. On the adaptive significance of leaf height in forest herbs. The American Naturalist 120: 353–381.

[CIT0033] Givnish TJ . 2020. The adaptive geometry of trees revisited. The American Naturalist 195: 935–947. 10.1086/708498 32469655

[CIT0034] Gurevitch J , Schuepp PH. 1990. Boundary layer properties of highly dissected leaves: an investigation using an electrochemical fluid tunnel. Plant, Cell & Environment 13: 783–792.

[CIT0035] Harper JL, Bell AD . 1979. The population dynamics of growth form in organisms with modular construction. In: Anderson RM, Turner BD, Taylor LR, eds. *Population dynamics” 20th symposium British ecological society*. Oxford, UK: Blackwell Publishing, 29–52.

[CIT0036] Heerema R , Weinbaum S, Pernice F, Dejong T. 2008. Spur survival and return bloom in almond [*Prunus dulcis* (Mill.) DA Webb] varied with spur fruit load, specific leaf weight, and leaf area. The Journal of Horticultural Science and Biotechnology 83: 274–281.

[CIT0037] Hikosaka K , Kumagai To, Ito A. 2016. Modeling canopy photosynthesis. In: Hikosaka K, Niinemets Ü, Anten NPR, eds. Canopy photosynthesis: from basics to applications. Dordrecht: Springer Netherlands, 239–268.

[CIT0038] Huang L , Niinemets U, Ma J, Schrader J, Wang R, Shi P. 2021. Plant age has a minor effect on non-destructive leaf area calculations in Moso Bamboo (*Phyllostachys edulis*). Symmetry 13: 369.

[CIT0039] Huang W , Su X, Ratkowsky DA, Niklas KJ, Gielis J, Shi P. 2019. The scaling relationships of leaf biomass vs. leaf surface area of 12 bamboo species. Global Ecology and Conservation 20: e00793.

[CIT0040] Huxley JS . 1932. Problems of relative growth. London: Methuen.

[CIT0041] Iwabe R , Koyama K, Komamura R. 2021. Shade avoidance and light foraging of a clonal woody species, *Pachysandra terminalis*. Plants 10: 809. 33924069 10.3390/plants10040809 PMC8074284

[CIT0042] Japan Meteorological Agency. 2020. https://www.jma.go.jp. Accessed 14 September 2020.

[CIT0043] John GP , Scoffoni C, Buckley TN, Villar R, Poorter H, Sack L. 2017. The anatomical and compositional basis of leaf mass per area. Ecology Letters 20: 412–425. 28198076 10.1111/ele.12739

[CIT0044] Kawai K , Okada N. 2020. Leaf vascular architecture in temperate dicotyledons: correlations and link to functional traits. Planta 251: 17. 10.1007/s00425-019-03295-z 31776668

[CIT0045] Kleiman D , Aarssen LW. 2007. The leaf size/number trade-off in trees. Journal of Ecology 95: 376–382.

[CIT0046] Komamura R , Koyama K, Yamauchi T, Konno Y, Gu L. 2021. Pollination contribution differs among insects visiting *Cardiocrinum cordatum* flowers. Forests 12: 452.

[CIT0047] Koyama K , Hidaka Y, Ushio M. 2012. Dynamic scaling in the growth of a non-branching plant, *Cardiocrinum cordatum*. PLoS One 7: e45317. 23028928 10.1371/journal.pone.0045317 PMC3446904

[CIT0048] Koyama K , Kikuzawa K. 2009. Is whole-plant photosynthetic rate proportional to leaf area? A test of scalings and a logistic equation by leaf demography census. American Naturalist 173: 640–649. 10.1086/597604 19275491

[CIT0049] Koyama K , Kikuzawa K. 2010. Geometrical similarity analysis of photosynthetic light response curves, light saturation and light use efficiency. Oecologia 164: 53–63. 20425123 10.1007/s00442-010-1638-9

[CIT0050] Koyama K , Shirakawa H, Kikuzawa K. 2020. Redeployment of shoots into better-lit positions within the crowns of saplings of five species with different growth patterns. Forests 11: 1301.

[CIT0051] Koyama K , Yamamoto K, Ushio M. 2017. A lognormal distribution of the lengths of terminal twigs on self-similar branches of elm trees. Proceedings of the Royal Society B: Biological Sciences 284: 20162395. 10.1098/rspb.2016.2395 PMC5247503 28053062

[CIT0052] Kurosawa Y , Mori S, Wang M, et al 2021. Initial burst of root development with decreasing respiratory carbon cost in *Fagus crenata* Blume seedlings. Plant Species Biology 36: 146–156.

[CIT0053] Kusi J , Karsai I. 2020. Plastic leaf morphology in three species of *Quercus*: The more exposed leaves are smaller, more lobated and denser. Plant Species Biology 35: 24–37.

[CIT0054] Laurans M , Vincent G. 2016. Are inter- and intraspecific variations of sapling crown traits consistent with a strategy promoting light capture in tropical moist forest? Annals of Botany 118: 983–996. 27489160 10.1093/aob/mcw140 PMC5055821

[CIT0055] Lecigne B , Delagrange S, Taugourdeau O. 2021. Annual shoot segmentation and physiological age classification from tls data in trees with acrotonic growth. Forests 12: 391.

[CIT0056] Lehnebach R , Beyer R, Letort V, Heuret P. 2018. The pipe model theory half a century on: a review. Annals of Botany 121: 773–795. 29370362 10.1093/aob/mcx194 PMC5906905

[CIT0057] Levionnois S , Salmon C, Alméras T, et al 2021. Anatomies, vascular architectures, and mechanics underlying the leaf size-stem size spectrum in 42 Neotropical tree species. Journal of Experimental Botany 72: 7957–7969. 34390333 10.1093/jxb/erab379

[CIT0058] Li Y , Niklas KJ, Gielis J, et al 2021. Anelliptical blade is not a true ellipse, but a superellipse–evidence from two *Michelia* species. Journal of Forestry Research.

[CIT0059] Lin S , Niklas KJ, Wan Y, et al 2020. Leaf shape influences the scaling of leaf dry mass vs. area: a test case using bamboos. Annals of Forest Science 77: 11.

[CIT0060] Lopes C , Pinto P. 2005. Easy and accurate estimation of grapevine leaf area with simple mathematical models. Vitis 44: 55–61.

[CIT0061] Maslova NP , Karasev EV, Xu S-L, et al 2021. Variations in morphological and epidermal features of shade and sun leaves of two species: *Quercus bambusifolia* and *Q. myrsinifolia*. American Journal of Botany 108: 1441–1463. 34431508 10.1002/ajb2.1706

[CIT0062] Meng F , Cao R, Yang D, Niklas KJ, Sun S. 2013. Within-twig leaf distribution patterns differ among plant life-forms in a subtropical Chinese forest. Tree Physiology 33: 753–762. 23933830 10.1093/treephys/tpt053

[CIT0063] Milla R . 2009. The leafing intensity premium hypothesis tested across clades, growth forms and altitudes. Journal of Ecology 97: 972–983.

[CIT0064] Miranda J , Finley J, Aarssen L. 2019. Leafing intensity predicts fecundity allocation in herbaceous angiosperms. Folia Geobotanica 54: 191–198.

[CIT0065] Mori S , Yamaji K, Ishida A, et al 2010. Mixed-power scaling of whole-plant respiration from seedlings to giant trees. Proceedings of the National Academy of Sciences of the United States of America 107: 1447–1451. 20080600 10.1073/pnas.0902554107 PMC2824365

[CIT0066] Niinemets Ü . 2016. Within-canopy variations in functional leaf traits: structural, chemical and ecological controls and diversity of responses. In: Hikosaka K, Niinemets Ü, Anten NPR, eds. Canopy photosynthesis: from basics to applications. Dordrecht: Springer Netherlands, 101–141.

[CIT0067] Niinemets U , Portsmuth A, Tena D, Tobias M, Matesanz S, Valladares F. 2007. Do we underestimate the importance of leaf size in plant economics? Disproportional scaling of support costs within the spectrum of leaf physiognomy. Annals of Botany 100: 283–303. 17586597 10.1093/aob/mcm107 PMC2735320

[CIT0068] Niklas KJ . 1994. Plant allometry: The scaling of form and process. Chicago: University of Chicago Press.

[CIT0069] Niklas KJ , Enquist BJ. 2001. Invariant scaling relationships for interspecific plant biomass production rates and body size. Proceedings of the National Academy of Sciences 98: 2922–2927. 10.1073/pnas.041590298 PMC30241 11226342

[CIT0070] Niklas KJ , Enquist BJ. 2002. On the vegetative biomass partitioning of seed plant leaves, stems, and roots. The American Naturalist 159: 482–497. 10.1086/339459 18707431

[CIT0071] Ogawa K . 2008. The leaf mass/number trade-off of Kleiman and Aarssen implies constancy of leaf biomass, its density and carbon uptake in forest stands: scaling up from shoot to stand level. Journal of Ecology 96: 188–191.

[CIT0072] Ogawa K , Furukawa A, Hagihara A, Abdullah AM, Awang M. 1995. Morphological and phenological characteristics of leaf development of *Durio zibethinus* Murray (Bombacaceae). Journal of Plant Research 108: 511–515.

[CIT0073] Oguchi R , Hikosaka K, Hirose T. 2005. Leaf anatomy as a constraint for photosynthetic acclimation: differential responses in leaf anatomy to increasing growth irradiance among three deciduous trees. Plant Cell and Environment 28: 916–927.

[CIT0074] Ohara M , Narumi T, Yoshizane T, Okayasu T, Masuda J, Kawano S. 2006. 7: *Cardiocrinum cordatum* (Thunb.) Makino (Liliaceae). Plant Species Biology 21: 201–207.

[CIT0075] Okie JG . 2013. General models for the spectra of surface area scaling strategies of cells and organisms: fractality, geometric dissimilitude, and internalization. American Naturalist 181: 421–439. 10.1086/669150 23448890

[CIT0076] Olson M , Rosell JA, Martínez-Pérez C, et al 2020. Xylem vessel-diameter–shoot-length scaling: ecological significance of porosity types and other traits. Ecological Monographs 90: e01410.

[CIT0077] Olson ME , Aguirre-Hernández R, Rosell JA. 2009. Universal foliage-stem scaling across environments and species in dicot trees: plasticity, biomechanics and Corner’s Rules. Ecology Letters 12: 210–219. 19141123 10.1111/j.1461-0248.2008.01275.x

[CIT0078] Olson ME , Rosell JA, Zamora Muñoz S, Castorena M. 2018. Carbon limitation, stem growth rate and the biomechanical cause of Corner’s rules. Annals of Botany 122: 583–592. 29889257 10.1093/aob/mcy089 PMC6153482

[CIT0079] Pearcy RW , Muraoka H, Valladares F. 2005. Crown architecture in sun and shade environments: assessing function and trade-offs with a three-dimensional simulation model. New Phytologist 166: 791–800. 15869642 10.1111/j.1469-8137.2005.01328.x

[CIT0080] Phinopoulos V , Cadima J, Lopes C. 2015. Estimation of leaf area in grapevine cv. Syrah using empirical models. In: 19th International Meeting of Viticulture GiESCO, Peach Rouge-Montpellier, 31 May–5 June, 2015, vol. 1, 385–388: GiESCO.

[CIT0081] R Core Team. 2021. R: a language and environment for statistical computing. Vienna: R Foundation for Statistical Computing.

[CIT0082] Royer DL , Wilf P, Janesko DA, Kowalski EA, Dilcher DL. 2005. Correlations of climate and plant ecology to leaf size and shape: potential proxies for the fossil record. American Journal of Botany 92: 1141–1151. 21646136 10.3732/ajb.92.7.1141

[CIT0083] Savage VM , Bentley LP, Enquist BJ, et al 2010. Hydraulic trade-offs and space filling enable better predictions of vascular structure and function in plants. Proceedings of the National Academy of Sciences of the United States of America 107: 22722–22727. 21149696 10.1073/pnas.1012194108 PMC3012458

[CIT0084] Schneider CA , Rasband WS, Eliceiri KW. 2012. NIH Image to ImageJ: 25 years of image analysis. Nature Methods 9: 671–675. 22930834 10.1038/nmeth.2089 PMC5554542

[CIT0085] Schrader J , Shi P, Royer DL, et al 2021. Leaf size estimation based on leaf length, width and shape. Annals of Botany 128: 395–406. 34157097 10.1093/aob/mcab078 PMC8414912

[CIT0086] Schuepp PH . 1993. Tansley Review No. 59 Leaf boundary layers. New Phytologist 125: 477–507. 33874584 10.1111/j.1469-8137.1993.tb03898.x

[CIT0087] Scott SL , Aarssen LW. 2012. Within-species leaf size–number trade-offs in herbaceous angiosperms. Botany 90: 223–235.

[CIT0088] Scott SL , Aarssen LW. 2013. Leaf size versus leaf number trade-offs in dioecious angiosperms. Journal of Plant Ecology 6: 29–35.

[CIT0089] Seleznyova AN , Greer DH. 2001. Effects of temperature and leaf position on leaf area expansion of kiwifruit (*Actinidia deliciosa*) shoots: development of a modelling framework. Annals of Botany 88: 605–615.

[CIT0090] Shi P-J , Li Y-R, Niinemets U, Olson E, Schrader J. 2021 *a*. Influence of leaf shape on the scaling of leaf surface area and length in bamboo plants. Trees 35: 709–715.

[CIT0091] Shi P , Li Y, Hui C, Ratkowsky DA, Yu X, Niinemets U. 2020. Does the law of diminishing returns in leaf scaling apply to vines? – Evidence from 12 species of climbing plants. Global Ecology and Conservation 21: e00830.

[CIT0092] Shi P , Yu K, Niinemets U, Gielis J. 2021 *b*. Can leaf shape be represented by the ratio of leaf width to length? Evidence from nine species of *Magnolia* and *Michelia* (Magnoliaceae). Forests 12: 41.

[CIT0093] Shinozaki K , Yoda K, Hozumi K, Kira T. 1964. A quantitative analysis of plant form-the pipe model theory: I. Basic analyses. Japanese Journal of Ecology 14: 97–105.

[CIT0094] Smith DD , Sperry JS, Adler FR. 2017. Convergence in leaf size versus twig leaf area scaling: do plants optimize leaf area partitioning? Annals of Botany 119: 447–456. 28028019 10.1093/aob/mcw231 PMC7296615

[CIT0095] Spann TM , Heerema RJ. 2010. A simple method for non-destructive estimation of total shoot leaf area in tree fruit crops. Scientia Horticulturae 125: 528–533.

[CIT0096] Sterck FJ , Schieving F. 2007. 3-D growth patterns of trees: effects of carbon economy, meristem activity, and selection. Ecological Monographs 77: 405–420.

[CIT0097] Sterck FJ , Schieving F, Lemmens A, Pons TL. 2005. Performance of trees in forest canopies: explorations with a bottom-up functional–structural plant growth model. New Phytologist 166: 827–843. 15869645 10.1111/j.1469-8137.2005.01342.x

[CIT0098] Sun J , Chen X, Wang M, Li J, Zhong Q, Cheng D. 2020. Application of leaf size and leafing intensity scaling across subtropical trees. Ecology and Evolution 10: 13395–13402. 33304546 10.1002/ece3.6943 PMC7713914

[CIT0099] Sun J , Fan R, Niklas KJ, et al 2017. ‘Diminishing returns’ in the scaling of leaf area vs. dry mass in Wuyi Mountain bamboos, Southeast China. American Journal of Botany 104: 993–998. 28701295 10.3732/ajb.1700068

[CIT0100] Sun J , Wang M, Lyu M, et al 2019 *a*. Stem and leaf growth rates define the leaf size vs. number trade-off. AoB PLANTS 11: plz063. 31777650 10.1093/aobpla/plz063 PMC6863467

[CIT0101] Sun J , Wang M, Lyu M, et al 2019 *b*. Stem diameter (and not length) limits twig leaf biomass. Frontiers in Plant Science 10: 185. 30846996 10.3389/fpls.2019.00185 PMC6393343

[CIT0102] Sun S , Jin D, Shi P. 2006. The leaf size–twig size spectrum of temperate woody species along an altitudinal gradient: an invariant allometric scaling relationship. Annals of Botany 97: 97–107. 16254019 10.1093/aob/mcj004 PMC2803375

[CIT0103] Sun S , Niklas Karl J, Fang F, Xiang S, Wu X, Yang X. 2010. Is branching intensity interspecifically related to biomass allocation? A survey of 25 dicot shrub species from an open-growing dry valley. International Journal of Plant Sciences 171: 615–625.

[CIT0104] Teobaldelli M , Basile B, Giuffrida F, et al 2019 *a*. Analysis of cultivar-specific variability in size-related leaf traits and modeling of single leaf area in three medicinal and aromatic plants: *Ocimum basilicum* L., *Mentha* spp., and *Salvia* spp. Plants 9: 13. 10.3390/plants9010013 PMC7020212 31861772

[CIT0105] Teobaldelli M , Rouphael Y, Fascella G, Cristofori V, Rivera CM, Basile B. 2019 *b*. Developing an accurate and fast non-destructive single leaf area model for loquat (*Eriobotrya japonica* Lindl) cultivars. Plants 8: 230. 10.3390/plants8070230 PMC6681347 31319530

[CIT0106] Teobaldelli M , Rouphael Y, Gonnella M, et al 2020. Developing a fast and accurate model to estimate allometrically the total shoot leaf area in grapevines. Scientia Horticulturae 259: 108794.

[CIT0107] Trueba S , Delzon S, Isnard S, Lens F. 2019. Similar hydraulic efficiency and safety across vesselless angiosperms and vessel-bearing species with scalariform perforation plates. Journal of Experimental Botany 70: 3227–3240. 30921455 10.1093/jxb/erz133

[CIT0108] Trueba S , Isnard S, Barthélémy D, Olson ME. 2016. Trait coordination, mechanical behaviour and growth form plasticity of *Amborella trichopoda* under variation in canopy openness. AoB PLANTS 8: plw068. 10.1093/aobpla/plw068 PMC5142121 27672131

[CIT0109] Valladares F , Brites D. 2004. Leaf phyllotaxis: Does it really affect light capture? Plant Ecology 174: 11–17.

[CIT0110] Vogel S . 2009. Leaves in the lowest and highest winds: temperature, force and shape. New Phytologist 183: 13–26. 19413689 10.1111/j.1469-8137.2009.02854.x

[CIT0111] Wang M , Mori S, Kurosawa Y, Ferrio JP, Yamaji K, Koyama K. 2021. Consistent scaling of whole-shoot respiration between Moso bamboo (*Phyllostachys pubescens*) and trees. Journal of Plant Research 134: 989–997. 34115233 10.1007/s10265-021-01320-5 PMC8364903

[CIT0112] Warton DI , Duursma RA, Falster DS, Taskinen S. 2012. smatr 3 – an R package for estimation and inference about allometric lines. Methods in Ecology and Evolution 3: 257–259.

[CIT0113] Warton DI , Wright IJ, Falster DS, Westoby M. 2006. Bivariate line-fitting methods for allometry. Biological Reviews 81: 259–291. 16573844 10.1017/S1464793106007007

[CIT0114] West GB , Enquist BJ, Brown JH. 2009. A general quantitative theory of forest structure and dynamics. Proceedings of the National Academy of Sciences of the United States of America 106: 7040–7045. 19363160 10.1073/pnas.0812294106 PMC2678466

[CIT0115] Westoby M , Wright IJ. 2003. The leaf size – twig size spectrum and its relationship to other important spectra of variation among species. Oecologia 135: 621–628. 16228258 10.1007/s00442-003-1231-6

[CIT0116] White PS . 1983. Corner’s rules in eastern deciduous trees: allometry and its implications for the adaptive architecture of trees. Bulletin of the Torrey Botanical Club 110: 203–212.

[CIT0117] Whitman T , Aarssen LW. 2010. The leaf size/number trade-off in herbaceous angiosperms. Journal of Plant Ecology 3: 49–58.

[CIT0118] Wickham H . 2016. ggplot2: elegant graphics for data analysis. Cham: Springer International Publishing.

[CIT0119] Wilke CO . 2016. *cowplot*: streamlined plot theme and plot annotations for ‘*ggplot2*’. CRAN Repos 2: R2.

[CIT0120] Xiang S , Liu Y, Fang F, Wu N, Sun S. 2009 *a*. Stem architectural effect on leaf size, leaf number, and leaf mass fraction in plant twigs of woody species. International Journal of Plant Sciences 170: 999–1008.

[CIT0121] Xiang S , Wu N, Sun S. 2009 *b*. Within-twig biomass allocation in subtropical evergreen broad-leaved species along an altitudinal gradient: allometric scaling analysis. Trees 23: 637–647.

[CIT0122] Xiang S , Wu N, Sun S. 2010. Testing the generality of the ‘leafing intensity premium’ hypothesis in temperate broad-leaved forests: a survey of variation in leaf size within and between habitats. Evolutionary Ecology 24: 685–701.

[CIT0123] Xu F , Guo W, Xu W, Wei Y, Wang R. 2009. Leaf morphology correlates with water and light availability: What consequences for simple and compound leaves? Progress in Natural Science 19: 1789–1798.

[CIT0124] Yagi T , Kikuzawa K. 1999. Patterns in size-related variations in current-year shoot structure in eight deciduous tree species. Journal of Plant Research 112: 343–352.

[CIT0125] Yan E-R , Wang X-H, Chang SX, He F. 2013. Scaling relationships among twig size, leaf size and leafing intensity in a successional series of subtropical forests. Tree Physiology 33: 609–617. 23824241 10.1093/treephys/tpt042

[CIT0126] Yang D , Li G, Sun S. 2008. The generality of leaf size versus number trade-off in temperate woody species. Annals of Botany 102: 623–629. 18682438 10.1093/aob/mcn135 PMC2701782

[CIT0127] Yang D , Li G, Sun S. 2009. The effects of leaf size, leaf habit, and leaf form on leaf/stem relationships in plant twigs of temperate woody species. Journal of Vegetation Science 20: 359–366.

[CIT0128] Yang D , Niklas KJ, Xiang S, Sun S. 2010. Size-dependent leaf area ratio in plant twigs: implication for leaf size optimization. Annals of Botany 105: 71–77. 19864268 10.1093/aob/mcp262 PMC2794065

[CIT0129] Yu X , Shi P, Schrader J, Niklas KJ. 2020. Nondestructive estimation of leaf area for 15 species of vines with different leaf shapes. American Journal of Botany 107: 1481–1490. 33169366 10.1002/ajb2.1560

[CIT0130] Zhu G , Niklas KJ, Li M, et al 2019. ‘Diminishing Returns’ in the scaling between leaf area and twig size in three forest communities along an elevation gradient of Wuyi Mountain, China. Forests 10: 1138.

